# Challenging Items on the ACE‐III: Insights from Cognitively Normal Older Adults in Kerala India

**DOI:** 10.1002/alz70857_104022

**Published:** 2025-12-25

**Authors:** Marnina B Stimmel, Emmeline Ayers, Joe Verghese, Erica Weiss

**Affiliations:** ^1^ Albert Einstein College of Medicine, Bronx, NY, USA; ^2^ Stony Brook University, Stony Brook, NY, USA

## Abstract

**Background:**

Valid cognitive screening tools are necessary as worldwide rates of dementia rise. Screening tools such as the Addenbrooke's Cognitive Examination‐III (ACE‐III) validated in several languages and countries, including India, are useful for identifying cognitive impairment in older adults. However, certain ACE‐III items may yield high error rates, even among cognitively normal individuals, especially when level of education is considered. This study assessed the performance of individual ACE‐III items in a cohort of cognitively normal older adults in India.

**Method:**

Participants included 132 Malayalam‐speaking older adults (mean age: 66.8, SD=4.7; 61% male; mean education: 10.6, SD=3.1 years) from the Kerala Einstein Study, a community‐based longitudinal study of pre‐dementia syndromes in Kerala, India. Participants completed the Malayalam‐version of the ACE‐III, along with a comprehensive neuropsychological battery. Cognitive normality was established through a consensus diagnosis by a neuropsychologist and neurologist; of these individuals 70 were without cognitive complaint and 62 reported some cognitive complaint. Frequencies of full, partial, and no credit for individual ACE‐III items were analyzed. Education was dichotomized to higher (10+ years) versus lower (<10 years) and chi square analyses of individual ACE‐III items between these groups were evaluated.

**Result:**

Certain ACE‐III items were nearly universally credited (≥94%) in this cognitively normal cohort, including orientation, word repetition, sentence repetition, comprehension of instructions, picture identification, word reading, dot counting, and letter identification. However, other items revealed high rates of partial or no credit, with over 25% of participants struggling to achieve perfect scores on tasks such as clock drawing, cube copying, picture naming, serial 7s, and recalling a name and address. For many of these items, lower education was significantly associated with higher error rates.

**Conclusion:**

While the ACE‐III is a robust and validated cognitive screening tool, certain items may have limited utility due to a high error rate among cognitively normal participants. This is particularly evident in Malayalam‐speaking Indian older adults with lower levels of education. These findings highlight that errors on specific ACE‐III items (as identified in this study) are relatively common in this population and should be interpreted cautiously to avoid mischaracterizing normal variations in performance as pathological.

